# Nephrotic syndrome and kidney failure due to immunocomplex-mediated renal damage in a patient with Waldenström's Macroglobulinemia: a case report

**DOI:** 10.1186/1757-1626-1-333

**Published:** 2008-11-19

**Authors:** Hector Castro, Rafael Valenzuela, Phillip Ruiz, Oliver Lenz, Mauricio Monrroy

**Affiliations:** 1 Department of Medicine, Division of General Internal Medicine, University of Miami/Jackson Memorial Medical Center, Miami, Florida, USA; 2 Pathology and Laboratory Medicine Service, Miami Veterans Affairs Medical Center, Miami, Florida, USA; 3 Immunopathology and University of Miami Transplant Laboratories, University of Miami/Jackson Memorial Medical Center, Miami, Florida, USA; 4 Department of Medicine, Division of Nephrology and Hypertension, University of Miami/Jackson Memorial Medical Center, Miami, Florida, USA

## Abstract

**Introduction:**

Unlike the quite frequent renal involvement seen in cases of Multiple Myeloma, the kidney is hardly ever compromised in patients with Waldenström's Macroglobulinemia. Nephrotic range proteinuria is a very unusual manifestation of renal injury in these patients and when present it is due to amyloid light-chain deposition most of the times.

**Case presentation:**

A 60-year-old male patient presented to the hospital with nephrotic syndrome, renal insufficiency, hypertension and lymphadenopathy. The investigations led to the diagnosis of Waldenström's Macroglobulinemia with associated nephrotic syndrome and chronic kidney disease due to an unusual form of hypocomplementemic glomerulopathy with histopathological features similar to those seen in mesangiocapillary glomerulonephritis type III, but lacking proliferative changes.

**Conclusion:**

We present an unusual case of immunologically-mediated renal damage in a patient with Waldenström's Macroglobulinemia, leading to non-amyloid nephrotic syndrome and chronic renal insufficiency.

## Introduction

Waldenström's Macroglobulinemia (WM) is a clonal B-cell lymphoproliferative disorder characterized primarily by bone marrow infiltration associated with IgM monoclonal gammopathy of any concentration in the serum [1]. It is a distinct clinicopathological entity and its underlying pathological diagnosis is Lymphoplasmacytic Lymphoma (LPL) [2]. LPL/WM is a rare lymphoid disorder accounting for only 1 to 2% of all hematologic malignancies [3]. Although several different pathogenic mechanisms that can affect multiple organs have been described in this disease [3], the kidney is usually spared as compared to Multiple Myeloma and the incidence of both renal failure and nephrotic syndrome is low [3-5]. This lesser degree of renal damage has been attributed to the lower frequency and severity of Bence-Jones proteinuria and hypercalcemia. In patients with coexisting WM and kidney disease, nephrotic-range proteinuria is seen in a small percentage of cases (7 – 28%) [6,7]. When present, the most common cause of nephrotic syndrome in these patients is AL amyloidosis [3,8,9]. Cases of WM with non-amyloid nephrotic syndrome are anecdotic in the worldwide literature.

## Case presentation

A 60-year old hispanic male presented to the hospital with a six-month history of slowly progressive bilateral lower extremity edema and weight gain. His past medical history was only significant for hypertension. On physical examination the patient had a blood pressure of 208/109 mmHg, a 4+ bilateral lower extremity edema up to the thighs, and multiple palpable non-tender axillary and inguinal lymph nodes, the rest of the exam was otherwise unremarkable.

The laboratory tests yielded the following relevant values: hemoglobin 100 g/L, hematocrit 30%, and absolute lymphocytosis (6.8 × 10^3^/μL). Urea nitrogen 19.6 mmol/L, creatinine 477 μmol/L, potassium 5.1 mmol/L, corrected calcium 2.3 mmol/L, albumin 16 g/L, total protein 40 g/L, cholesterol 6.72 mmol/L, phosphate 2.32 mmol/L, intact parathyroid hormone 360 ng/L; high erythrocyte sedimentation rate (119 mm/hr); microscopic hematuria (37 rbc/hpf) and nephrotic range proteinuria (10.3 g/24 h); ANA, ANCA, anti-DNAds and anti-GBM negative; low complement levels (C3 0.15 g/L and C4 0.02 g/L) and absent cryoglobulins. Normal IgA, low IgG (3.01 g/L) and increased IgM (9.63 g/L). Negative HBsAg, anti-HCV and HIV. Serum protein electrophoresis revealed a monoclonal spike (0.33 g/dl) and the immunofixation electrophoresis showed a monoclonal gammopathy IgM κ.

On retroperitoneal ultrasound the right kidney measured 12.4 cm, the left kidney 12.3 cm, and both showed echogenic cortex. A CT scan of the thorax, abdomen and pelvis reported multiple axillary, pelvic and retroperitoneal lymphadenopathy.

### Lymph node biopsy

Abundant Congo red negative hyaline-like material was observed. Small lymphocytes with plasmacytoid appearance were identified. The lymphocytoid population was CD20+ and CD79a+ representing B cells, and it was negative for CD3, CD5, CD23, lambda and kappa. IgA, IgG and IgM immunoreactive cells were observed.

The bone marrow biopsy showed hypercellularity due to normoblastic erythroid hyperplasia consistent with a solitary large parenchymal lymphoid aggregate of small lymphocytes. The immunophenotype was consistent with an atypical B cell population and plasma cells were present by flow cytometry.

### Kidney biopsy

**Figure 1:**
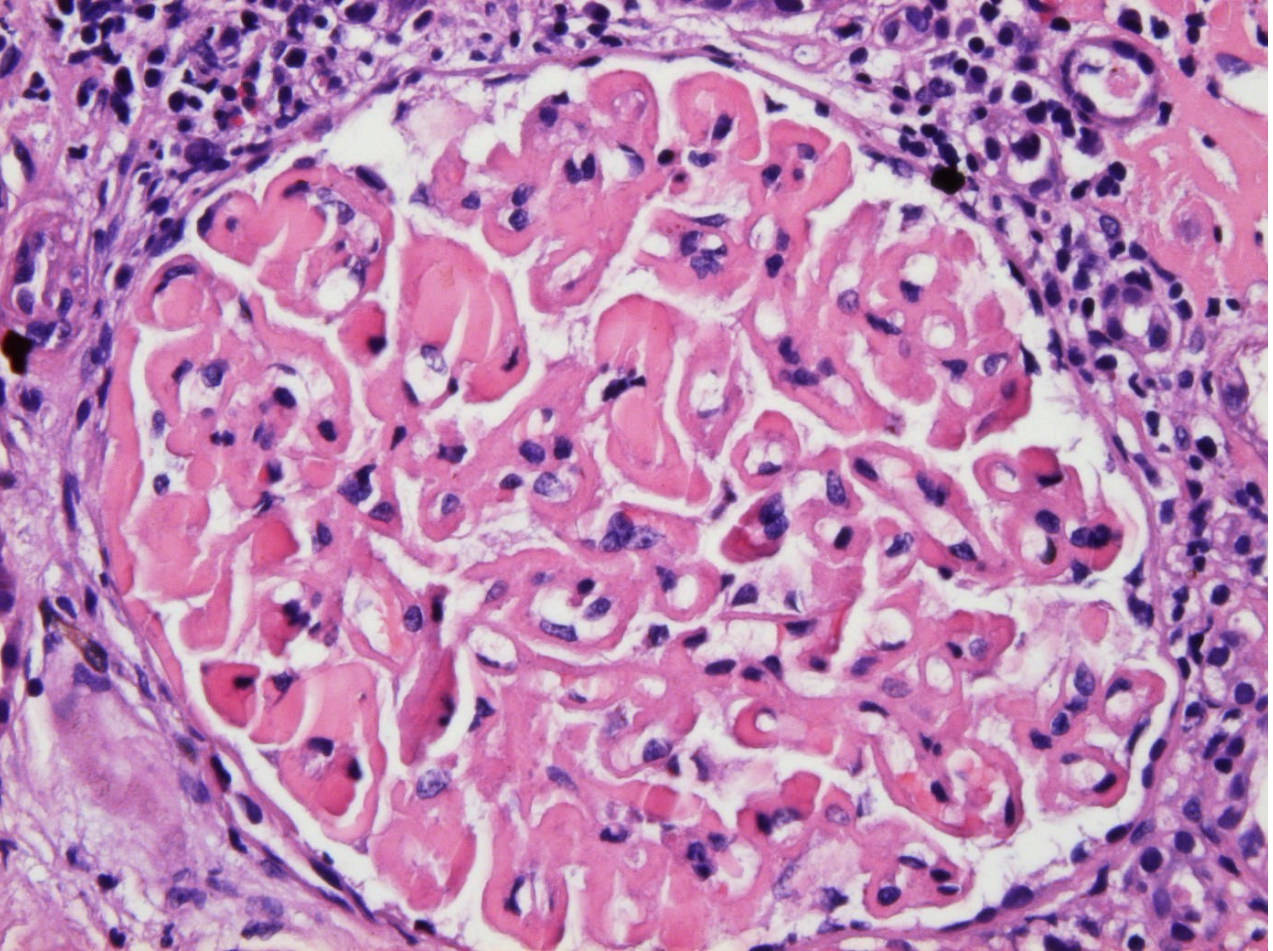
**Light Microscopy**. There is marked, global, homogeneous, eosinophilic thickening of the glomerular basement membrane with segmental accentuation. Homogeneous, eosinophilic globules are seen in the lumen of occasional capillary loops. The capillary lumina appear reduced in diameter but no inflammatory or proliferative changes are observed. The periglomerular interstitial space shows lymphocytic infiltration. Focal interstitial deposition of homogeneous eosinophilic material is present in the right upper corner of the picture (H&E × 400).

**Figure 2:**
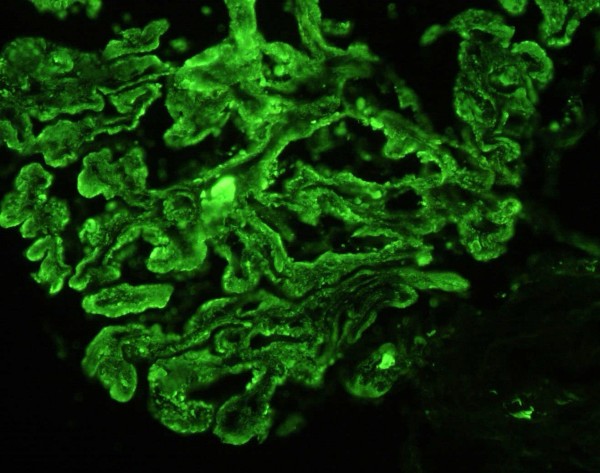
**Immunofluorescence**. Global granular and homogeneous deposition of IgG along the glomerular basement membrane. Notice the presence of IgG containing globules in rare capillary loops. They seem to correspond to the eosinophilic globules seen by light microscopy and large electron dense deposits detected by electron microscopy (FITC labeled anti-IgG × 400).

**Figure 3:**
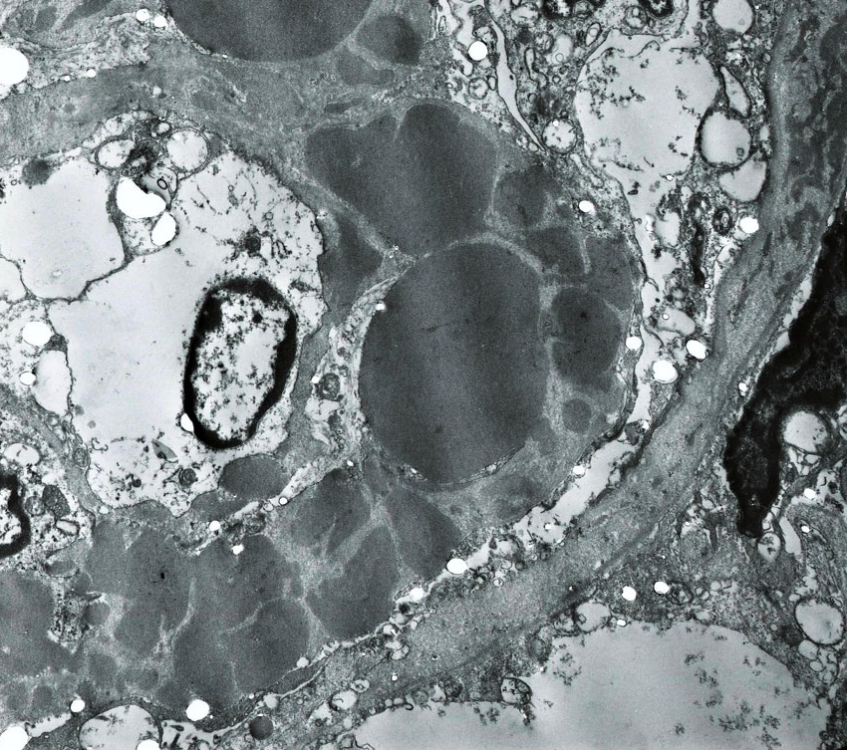
**Electron Microscopy**. There is marked thickening of the glomerular basement membrane due to the presence of numerous electron dense deposits located at different levels. The deposits vary in size, tend to be spherical in shape and blend together. Under higher magnifications, they did not exhibit a fibrillary or micro-tubular substructure. Notice a thin subendothelial layer of duplicated basement membrane, also containing electron dense deposits, with cellular interposition. The capillary lumen appears significantly reduced in diameter. Also notice electron dense deposits present in the basement membrane of Bowman's capsule on the right upper corner (Uranyl acetate & lead citrate × 35,000).

**Figure 4:**
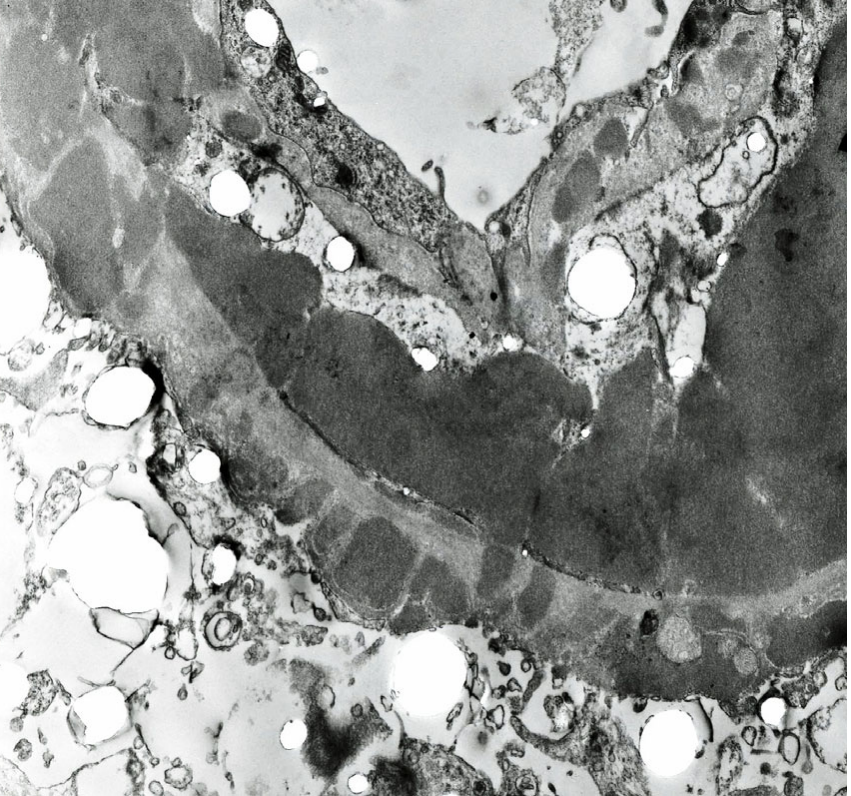
**Electron Microscopy**. This field illustrates a large subendothelial and several, much smaller, subepithelial electron dense deposits. This pattern is similar to that originally described in MPGN type III and also often seen in proliferative lupus GN. Notice the duplication of the glomerular basement membrane with cellular interposition. The duplicated segment also contains electron dense deposits. Occasionally giant, subendothelial, globular electron dense deposits reduced the capillary loop to a pin-point lumen. Probably they correspond to the globules seen by light and fluorescence microscopy (Uranyl acetate and lead citrate × 40,000).

On light microscopy 11 of 18 glomeruli were globally sclerosed and more than 50% of the specimen showed interstitial fibrosis. Marked, global and homogeneous thickening of the glomerular basement membrane, with segmental accentuation due to a strongly PAS positive eosinophilic material was seen and it was Congo red negative (Figure 1). There were no proliferative or inflammatory changes. Focal nodular collections of the same material were present in the tubulointerstitial areas and lymphocytic infiltration was present in the periglomerular interstitial space. Immunofluorescence showed a diffuse global, granular to homogenous deposition of IgG (3+), IgA (3+), IgM (2+), C3 (3+), C4 (3+), C1q (3+), albumin (3+), kappa (3+), lambda (3+) and fibrinogen (1+) involving principally the basement membranes (Figure 2). Focal homogenous deposition of the same immunoreactants with similar fluorescence intensity was seen in the tubulointerstitial areas. No vascular fluorescence was apparent. Finally, electron microscopy showed extensive glomerular sclerosis and basement membrane thickening with significant reduction of the capillary lumen in the remaining glomeruli (Figure 3). There were numerous electron-dense deposits present in both mesangium and glomerular membranes, but principally in the latter. Some of them clearly showed a subendothelial or subepithelial location (Figure 4). The deposits did not exhibit an organized substructure (microtubular or fibrillary). No fibrin thrombi were found. The interstitial areas were expanded due to the presence of mononuclear inflammatory cells (monocytes and plasma cells), increased collagen, and electron dense deposits similar to those identified in glomeruli.

The patient was started on β-blocker and calcium channel blocker therapy for blood pressure control, Erythropoietin for anemia treatment, phosphate binder and vitamin D analog for mineral and bone disorder management, loop diuretic for volume overload treatment and subcutaneous Heparin for thromboembolic prophylaxis; no renal replacement therapy was warranted during his hospital stay. The renal function worsened over the first days of his hospitalization with a peak creatinine of 583 μmol/L, hence, in light of the laboratory and preliminary histopathologic results, a trial of steroid therapy with Prednisone 1 mg/kg/day was started at that moment in order to avoid further kidney damage with a partial positive response over the following days (decrease in creatinine to 424 μmol/L). Further immunosuppressive therapy was considered at that point, unfortunately a post-surgical infection developed following the left inguinal lymph node biopsy leading to septic shock, and in spite of aggressive management with broad-spectrum antibiotic therapy, respiratory support, fluids and vasopressors the patient expired 22 days after his hospital admission. The autopsy report did not add any pertinent information to the case.

## Discussion

The diagnosis of Waldenström's Macroglobulinemia has always been challenging and has evolved from the original description of a clinical syndrome to the more recent designation as a distinct clinicopathologic entity [2]. Although significant advances have been made regarding lymphoma classification, there are no disease-defining morphological, immunophenotipic or chromosomal abnormalities specific for WM, a diagnosis can therefore be made on the basis of clinical and pathological findings [1]. We present a patient who was found to have an IgM monoclonal gammopathy in the serum associated with lymph node and bone marrow infiltration by a small B-cell population with plasmacytic differentiation. The lymph node biopsy revealed the presence of plasmacytoid cells that expressed an immunophenotypic profile consistent with WM according to the definition criteria [1]. The pattern of infiltration in the bone marrow was not intertrabecullar as defined by the diagnostic criteria; nevertheless, although this characteristic is considered to be helpful supporting evidence it is not the only described infiltrative pattern in cases of WM.

Renal involvement in WM is usually expressed as mild non-selective proteinuria and microscopic hematuria [9]. The renal compromise in our patient presented as microscopic hematuria, nephrotic syndrome and chronic renal insufficiency. The nephrotic syndrome work-up was only positive for significant hypocompletemia suggesting an immunologically-mediated disorder with serum complement consumption. Amyloidosis and cryoglobulinemia were ruled out. As mentioned above, nephrotic syndrome is a rare manifestation of kidney involvement in patients with WM; in a recent case series study only 2 out of 7 patients with coexisting WM and kidney disease presented with nephrotic-range proteinuria [7]. When present, this syndrome is usually caused by amyloidosis [3,8,9]; other very unusual causes include cryoglobulinemia [10,11], minimal change disease [12,13], intracapillary monoclonal deposit disease [7,14] and immunocomplex-mediated glomerulonephritis [15,16].

The usual renal histopathologic finding in WM is the presence of amorphous PAS-positive subendothelial deposits that usually occlude the capillary lumen and by electron microscopy consist of non-amyloid fibrillary material [6]. Immunofluorescence demonstrating the presence of IgM within the glomeruli is the most commonly described pattern [3,9,10,14,17].

The histopathologic findings in our patient are similar to those originally described in MPGN type III (but lacking proliferative changes) and also often seen in proliferative lupus nephritis, but they differ from the typical pattern of renal involvement in WM in several features, firstly the absence of thrombotic deposits in the capillary lumen, secondly no fibrillary material was identified in the electron microscopy, thirdly the presence of glomerular deposits consisting of multiple immunoglobulins and complement factors instead of IgM alone, and finally the simultaneous involvement of the tunulointerstitial areas. These findings can be attributed to an immunologically mediated mechanism with deposition of immune complex and complement factors. Furthermore, the evidence of hypocomplementemia correlates well with this suggested renal insult. Similar glomerular immunofluorescence characteristics have been described in only two previous reported cases however neither had renal impairment [15,16]. Even though the link between hematologic malignancies and glomerulonephritis has been well documented, the underlying pathogenesis in cases of WM is still unclear but it can be explained by the known intrinsic autoantibody activity of IgM [3].

In summary, we present a rare case of immunologically-mediated glomerulopathy associated with tubulointerstitial damage, leading to nephrotic syndrome and chronic kidney disease in a patient with LPL/WM.

## Consent

Written informed consent was obtained from the patient's next of kin for publication of this case report and accompanying images. A copy of the written consent is available for review by the Editor-in-Chief of this journal.

## Competing interests

The authors declare that they have no competing interests.

## Authors' contributions

HC collected the data and was the major contributor in writing the manuscript. RV and PR performed the histological examination of the kidney, RV described the final kidney biopsy pictures. OL revised the manuscript. MM collected and analyzed the clinical data. All authors read and approved the final manuscript.
